# Tenofovir-Induced Fanconi Syndrome Presenting with Life-Threatening Hypokalemia: Review of the Literature and Recommendations for Early Detection

**DOI:** 10.3390/jcm12227178

**Published:** 2023-11-20

**Authors:** Efstathia Liatsou, Ioanna Tatouli, Andreas Mpozikas, Maria-Markella Pavlou, Hariklia Gakiopoulou, Ioannis Ntanasis-Stathopoulos, Maria Gavriatopoulou, Sofoklis Kontogiannis, Meletios Athanasios Dimopoulos

**Affiliations:** 1 Department of Clinical Therapeutics, Alexandra Hospital, School of Medicine, National and Kapodistrian University of Athens, 11528 Athens, Greece; fayliatsou1@gmail.com (E.L.); ioannatatouli@gmail.com (I.T.); 90andreas@windowslive.com (A.M.); maria-markella_93@live.com (M.-M.P.); mgavria@med.uoa.gr (M.G.); agupiela@gmail.com (S.K.); 2 1st Department of Pathology, School of Medicine, National and Kapodistrian University of Athens, 11527 Athens, Greece; chgakiop@med.uoa.gr

**Keywords:** hepatitis B, tenofovir disoproxil, hypokalemia, acute kidney injury, Fanconi syndrome

## Abstract

Tenofovir disoproxil fumarate (TDF) is a nucleotide reverse transcriptase inhibitor that has been widely used for the treatment of patients with human immunodeficiency virus (HIV) and hepatitis B virus (HBV) infections. Despite the excellent safety records of this regimen, a few cases of acute renal failure and Fanconi syndrome have been reported among HIV patients exposed to TDF. However, in the HBV monoinfection scenario, only five cases of TDF-associated Fanconi syndrome have been reported thus far, two of them providing a confirmatory kidney biopsy. Here, we describe the case of a 68-year-old woman with chronic hepatitis B (CHB) who developed TDF-induced Fanconi syndrome that reverted after TDF withdrawal from tenofovir alafenamide. Though the overall risk of TDF-associated severe renal toxicity in HBV patients appears to be negligible, both glomerular and tubular functions should be monitored in patients exposed to TDF.

## 1. Introduction

The evolution of antiviral medications has led to significant progress in hepatitis B management [1]. During the last 10 years, five medications have received FDA approval for chronic hepatitis B, among which are tenofovir disoproxil fumarate and its prodrug tenofovir alafenamide [2,3]. Tenofovir is an acyclic adenine nucleotide analogue that interferes with HBV DNA polymerase [4]. After oral absorption, it is converted intracellularly via hydrolysis to tenofovir and then phosphorylated to the active tenofovir diphosphate [4]. A range of 20–30% of the drug is actively transported into renal proximal tubule cells by organic anion transporters and then the drug is secreted to the tubular lumen by the apical membrane transporters MRP-4 and MRP-2 encoded by ABCC4 and ABCC2 genes [4]. While tenofovir is mainly associated with adverse events from the gastrointestinal tract, post-marketing evaluations have outlined events of kidney toxicity with a vast spectrum of clinical manifestations, including acute kidney injury (AKI), chronic kidney disease (CKD) and futures of proximal tubulopathy, such us Fanconi syndrome, hypophosphatemia and decreased bone mineral density [5].

As its chemical structure resembles that of adefovir and cidofovir, agents with proved nephrotoxicity, cautiousness has risen regarding renal function in patients receiving the first treatment [6]. In fact, there have been reports describing cases of renal impairment in patients under tenofovir treatment; however this has not been supported by clinical trials, possibly due to selection bias [7]. More specifically, the clinical syndromes include proximal tubular dysfunction with or without GFR impairment, presented with complete or partial Fanconi syndrome, accompanied by renal tubular acidosis, glycosuria with normoglycemia, aminoaciduria, hypophosphatemia, hypouricemia and tubular proteinuria [6]. Fanconi syndrome is a metabolic disorder characterized by the damage of proximal renal tubular cells leading to the impaired proximal tubular reabsorption of amino acids, glucose, phosphate, urate and bicarbonate. Discrimination between partial and complete Fanconi syndrome depends on the severity of molecule malabsorption. As regards renal impairment, GFR is the abbreviation for glomerular filtration rate, and it is defined as the volume of plasma that is filtered by the glomeruli per unit of time. The measurement of GFR is based on the urinary or plasma clearance (https://www.sciencedirect.com/topics/medicine-and-dentistry/plasma-clearance, accessed on Monday, 5 June 2023) of an ideal or near-ideal exogenous marker such as inulin (https://www.sciencedirect.com/topics/agricultural-and-biological-sciences/inulin, accessed on Monday, 5 June 2023). Most data regarding epidemiology and clinical course are derived from the case series of HIV patients under tenofovir-based combination therapy [8]. More specifically, renal tubulopathy has been described in approximately 1–6% of patients who receive combination therapy with tenofovir disoproxil; however, confounding factors such as special epidemiological characteristics, comorbidities and chronic multiple-drug therapy make the causal correlation of tenofovir with nephrotoxicity rather complex [8]. Acute kidney injury may present 3–6 months after tenofovir initiation, and its clinical spectrum may extend from a mild decrease in GFR to hemodialysis dependence; however, this may reverse with drug discontinuation [9]. The duration of tenofovir administration has been correlated with the frequency and severity of tubulopathy symptoms. A study by Tan et al. demonstrated that the incidence of renal impairment at weeks 144 and 168 was higher in comparison with that at week 12 (OR: 4.1, 95% CI: 2.0–8.3 and OR: 8.2, 95% CI: 4.2–16.0, respectively) [10]. Those findings remained statistically significant after subgroup analysis for age and BMI [10].

Under the frame of nephroprotection and osteoporosis prevention, current clinical practice recommends the use of tenofovir alafenamide, a small-molecular-weight oral prodrug of tenofovir, TAF [11]. Special pharmacokinetic characteristics lead to lower tenofovir plasma concentrations, causing fewer kidney and bone adverse effects, with no impact on efficacy [11]. While most data on tenofovir fumarate are derived from the case series of patients with HIV infection under HAART, we present the case of a 68-year-old woman with a twenty-year history of tenofovir disoproxil fumarate treatment for hepatitis B who presented with anorexia, fatigue and severe non-anion gap metabolic acidosis, along with acute kidney injury.

## 2. Detailed Case Presentation

A 68-year-old woman visited our clinic with symptoms of non-bloody diarrhea, anorexia and fatigue for fifteen days. She had a medical history of severe osteoporosis and persisting bone pain, for which she received calcium and cholecalciferol, for which she had undergone thorough workup with a CT scan and a PET-CT that showed abnormal bone density indicative of osteoporosis non-relative to carcinomatosis. She had been diagnosed with chronic hepatitis B of an unknown time of onset that was being treated with tenofovir disoproxil. One year ago, she was hospitalized with acute renal injury due to episodes of vomiting and NSAID consumption for chronic pain. On her admission into our clinic, she was on treatment with tenofovir disoproxil 300 mg once daily, vitamin D supplements and NSAIDs.

On physical examination, she appeared to have mild abdominal pain. Her blood pressure was 120/75 mmHg, heart rate was 91 bpm and body temperature was 36.8 °C. The rest of the physical examination showed unremarkable findings. On blood tests, leukocytosis with an increased neutrophil count was noted (WBC: 26.800/μL: neutrophils: 98%/lymphocytes: 1.2%/eosinophils: 0.1%/monocytes: 0.6%), along with severe hypokalemia (K^+^: 1.8 mmol/L) and elevated serum creatinine (Cr: 2.9 mg/dL, eGFR: 18 mL/min). The rest of the laboratory exams showed unremarkable findings (Table 1). The arterial blood gas showed metabolic acidosis with a pH of 7.11, pO_2_ of 113 mmHg, pCO_2_ of 18 mmHg, HCO_3_ of 6.6 mmol/L and anion gap of 12. Due to symptoms of diarrhea and the recent consumption of amoxicillin–clavulanate and clarithromycin, fecal samples were tested for microbial infection. A Cl. difficile assessment and a fecal multiple-film array, which turned out negative, were carried out. Further investigation of diarrhea syndrome was carried out with the evaluation of antibodies against gliadin, endomysial and transglutaminase, serum aldosterone, renin, ACTH, cortisol, gastrin and VIP levels. The workup for celiac disease or endocrinopathy was negative. Spot urinalysis showed urine of pH 5.5, a specific gravity of 1020, protein (+) and ketone (+2). Urine sodium was 85 mmol/L, potassium was 87 mmol/L, and chloride was 86 mmol/L, with a urine anion gap ([Na^+^_u_ + K^+^]_u_ − [Cl^−^]_u_) of 86 mmol/L. Urine calcium and phosphorus were 47 mg/dL and 47.6 mg/dL, respectively. Calculated urine osmolality (2 × [Na + K]) + [urea nitrogen in mg/dL]/2.8 + [glucose in mg/dL]/18) was 939 mOsm/kg, with an approximate urine osmolal gap of 61 mOsm/kg. The 24 h urine protein levels were elevated at 2178/24 h, but urine protein immunofixation was negative. A renal ultrasound revealed normal bilateral echogenicity and kidney size was within a normal range (right: 10 mm; left: 12 mm).

The patient underwent hemodialysis due to hypokalemia-induced ECG abnormalities with ventricular premature complexes and U waves, and severe metabolic acidosis resistant to intravenous potassium, which resolved after 24 h of continuous renal replacement therapy. Non-anion gap metabolic acidosis was considered to be the cause of her presenting symptoms and extensive workup was performed for secondary renal tubular acidosis. Blood serum was tested for hepatitis A, B and C, rheumatoid factor, ANA, anti-sm, dsDNA, C-ANCA, P-ANCA, C3, C4, anti-Ro, anti-La, anti RNP, b2 microglobulin, and quantitative and qualitative immunoglobulin assays, as well as cancer biomarkers (AFP, CEA, Ca 19–9, Ca 125, and Ca 15–3). To exclude possible vitamin D toxicity, the levels of vitamin D and PTH were also evaluated, which turned out negative. The patient underwent upper GI endoscopy which showed abnormal gastric folding and mucus membrane atrophy of the biopsies that were obtained, indicative of chronic gastritis. A bone marrow biopsy was also performed which revealed no major pathology. The pharmacological history of the patient was re-evaluated meticulously and tenofovir was found among the routinely received drugs. Therefore, we decided to discontinue tenofovir as no other underlying cause had been identified. The patient’s symptoms resolved gradually and the clinical improvement was followed by the normalization of blood pH, electrolytes and renal function tests over the next two weeks. (Cr: 0.69 mg/dL, GFR: 74 mL/min, U: 40 mg/dL.)

**Figure 1:**
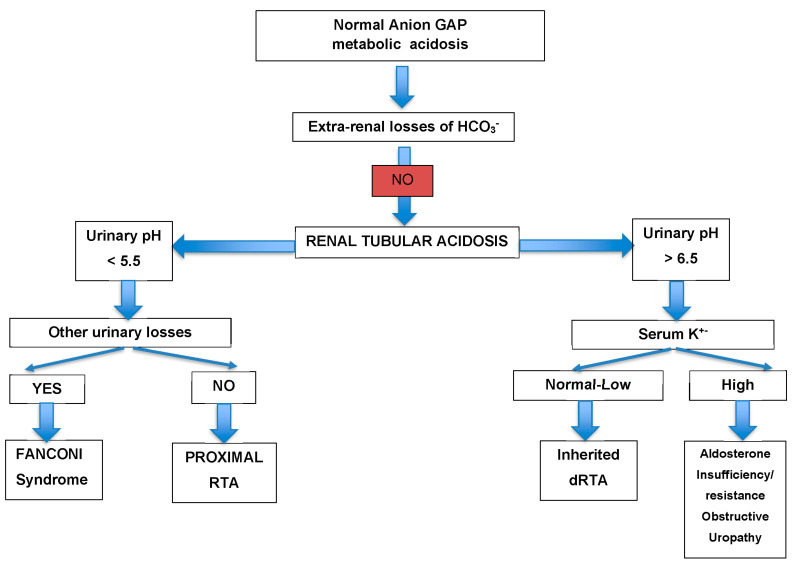
Algorithm for differential diagnosis of non-anion gap metabolic acidosis.

**Figure 2:**
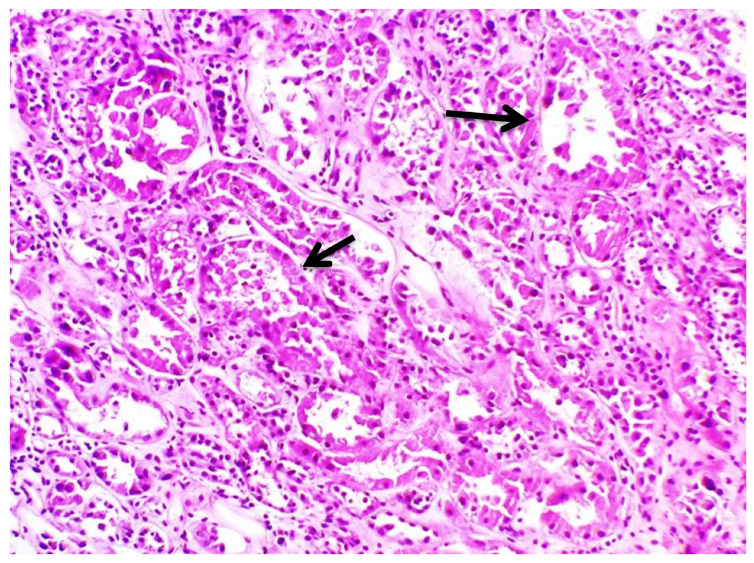
Diffuse and severe acute tubular injury (HE × 100).

**Figure 3:**
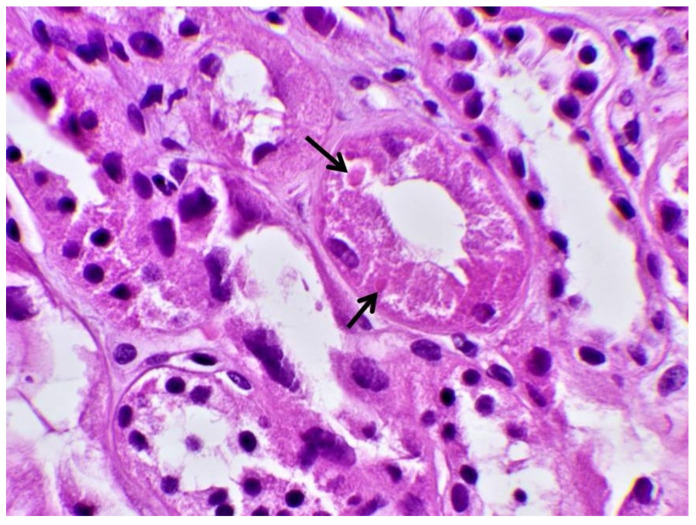
Proximal tubule with intracytoplasmic eosinophilic inclusions (black arrows) (HE × 400).

**Figure 4:**
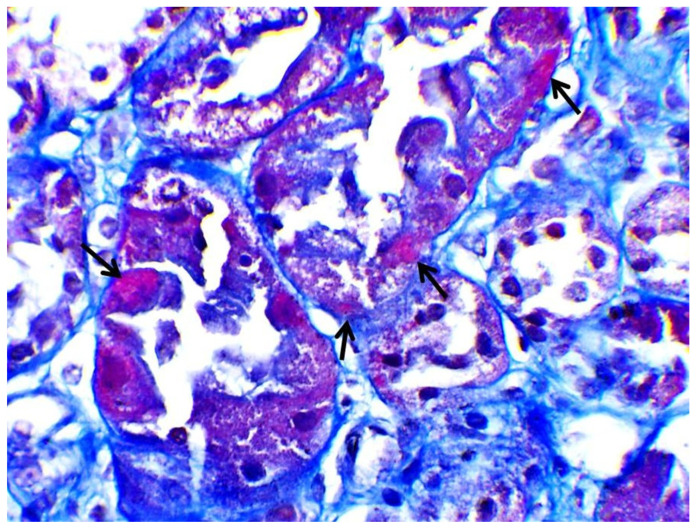
Proximal tubules with intracytoplasmic red/fuchsinophilic inclusions with Masson’s trichrome stain (black arrows) (×400).

**Figure 5:**
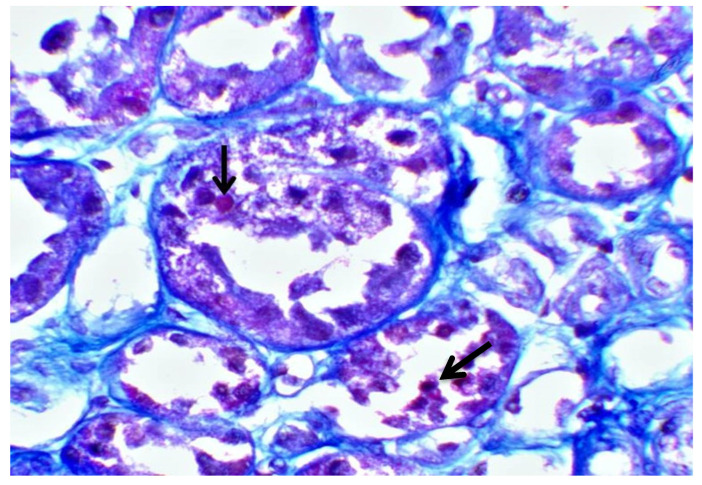
Proximal tubules with cytoplasmic swelling, focal desquamation with the segmental denudation of the underlying basement membrane, and an intracytoplasmic red inclusion (black arrow) (Masson × 400).

**Figure 6:**
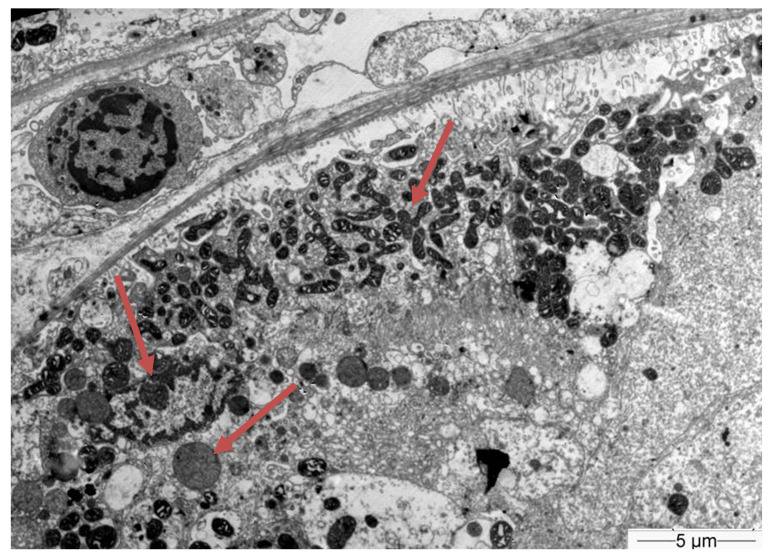
Electron microscope at a low magnification (×3500) showing the wide range of the size and shape of mitochrondria within proximal tubular epithelial cells.

**Figure 7:**
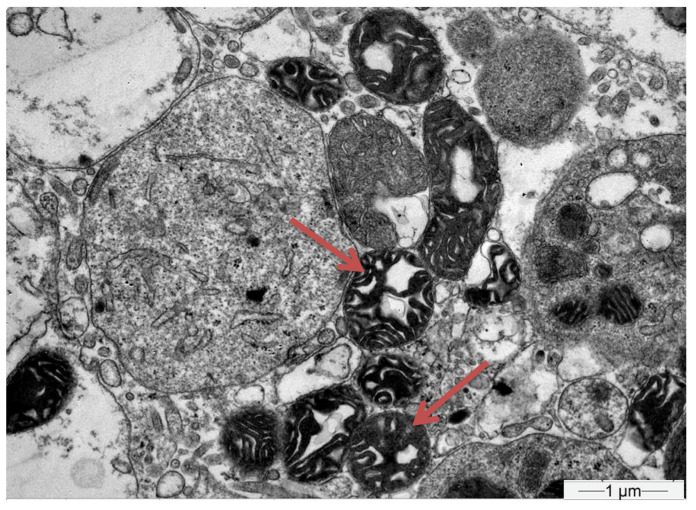
Electron microscope at a higher magnification (×14,000); red arrows show mitochondria with abnormal cristae and markedly enlarged mitochondria devoid of cristae are observed.

Subsequently, a renal biopsy was performed to confirm the diagnosis. The renal biopsy was examined as follows: (a) under a light microscope using the standard histochemical stains (pas, —Masson’s trichome, silver methenamine and Congo Red), (b) under an immunofluorescence microscope (with antibodies against IgG, IgA and IgM globulins, C3, C1q and C4 complement fractions, κ and light chains, and albumin and fibrinogen), and (c) under an electron microscope (Figure 1). The glomeruli were found to be normal (Figure 2) [7]. The renal biopsy demonstrated diffuse and severe acute proximal tubular injury without remarkable interstitial inflammation (Figure 2). The proximal tubules showed enlarged epithelial cells with occasional vacuolization and focal epithelial desquamation, with the segmental denudation of the underlying basement membranes. Intracytoplasmic inclusions within proximal tubular epithelial cells, red or fuchsinophilic with Masson’s trichrome stain, were also observed, raising the suspicion of giant mitochondria, a characteristic feature of tenofovir nephrotoxicity (Figure 3, Figure 4 and Figure 5) [7]. Indeed, an ultrastructural examination demonstrated mitochondrial abnormalities concerning the number, size and structure of mitochondria in the cytoplasm of proximal tubular cells (Figure 6 and Figure 7) [7]. Mitochondria with abnormal cristae or devoid of cristae, as well as markedly enlarged mitochondria, were seen, sometimes adjacent to normal-appearing mitochondria. Overall, these findings led to the diagnosis of tenofovir-induced Fanconi syndrome.

Following the positive biopsy results, the patient had to discontinue tenofovir disoproxil for HBV treatment at day 10 of her hospitalization, so she switched to tenofovir alafenamide, which is less commonly associated with Fanconi syndrome. After a follow-up of 8 months, kidney function remained within the normal range (Cr: 0.80 mg/dL; eGFR: 64 mL/min).

## 3. Discussion

Tenofovir-induced renal tubular acidosis is a rare complication of renal toxicity [12]. Data from post-marketing safety studies including 10,343 HIV-positive patients showed that serious renal adverse events were observed in 0.5% of patients receiving tenofovir during the last four years [12]. More specifically, Fanconi syndrome with symptoms of glycosuria, proteinuria and hypophosphatemia was seen in <0.1% of the studied population [12]. Adverse events were associated with patients’ advanced age, pre-existing elevated serum creatinine, low body weight, concomitant nephrotoxic medication and lower CD4 cell count [12]. The distinguishing histopathological findings, namely the presence of giant mitochondria identifiable as eosinophilic inclusions in the cytoplasm of proximal tubular cells under a light microscope, were first described by Herlitz LC et al. [7]. In their study, Herlitz et al. described a spectrum of mitochondrial abnormalities concerning the number, size, shape and crystal patterns reminiscent of changes observed in mitochondrial DNA depletion syndromes [7]. According to the authors, tenofovir toxicity primarily targets mitochondria and, as a result, pathological changes are most prominent in proximal tubules, leading to histological findings of toxic acute tubular injury and clinical findings of proximal tubular dysfunction with Fanconi syndrome [7]. In our case, the histopathological evaluation of the kidney tissue revealed large mitochondria in the cytoplasm of enlarged proximal tubular epithelial cells with occasional vacuolization and focal epithelial desquamation, with the segmental denudation of the underlying basement membranes.

The first case report of tenofovir-associated Fanconi syndrome was presented by Verhelst et al., describing a 45-year-old woman with hepatitis C and HIV infection under tenofovir treatment who presented with polyuria, hypokalemia and acute renal failure [13]. Since then, 19 cases have been reported in HIV-positive patients receiving tenofovir in combination with other antiretroviral agents such as ritonavir/lopinavir in 16 cases, drugs that enhance the proximal tubular intracellular accumulation of tenofovir and hence Fanconi syndrome. Low creatinine levels were observed in 16 out of 19 patients due to the patients’ low body weight, and creatinine clearance has been proposed as a better parameter for the evaluation of renal function. The time lapse between the initiation of tenofovir and renal abnormalities varied from 1 to 26 months. More specifically, in a series of seven similar cases presented by Peyrere et al., signs and symptoms of renal impairment appeared 5 weeks to 16 months from drug initiation [14].

A prospective study by Labarga P. et al. enrolled patients with HIV infection receiving HAART and evaluated their renal function and proximal tubular dysfunction between active TDF treatment and TDF-naive patients [15]. Tubular dysfunction was more frequent in patients receiving TDF (22%) in comparison with that in tenofovir disoproxil fumarate-naive patients (6%) and patients never exposed to antiretrovirals (12%) [15]. This dysfunction was mostly demonstrated with a lower fractional phosphorus reabsorption fraction, and complete Fanconi syndrome was identified in three cases, all under treatment with tenofovir [15]. Further univariate and multivariate analysis showed that factors such as age, diabetes, exposure to other nephrotoxic drugs and lower CD4 cell count were associated with tubular damage [15]. Rodriguez-Novoa et al. evaluated kidney dysfunction and markers of tubulopathy in 116 patients with HIV receiving tenofovir-containing therapy in a single institution [16]. Among them, 19 patients fulfilled the diagnostic criteria of tubulopathy, and different clinical and biochemical parameters were further evaluated as predisposing factors for tenofovir nephrotoxicity, such as older age, low BMI and mutations in ABCC2 gene polymorphisms that encode proteins implicated in tenofovir efflux at the luminal surface of tubular cells. Woodward et al. included 22 HIV patients who had been referred for renal toxicity primarily associated with tenofovir therapy. Among different markers of renal function, eGFR alone is not recommended for renal toxicity screening as some patients presented with isolated proteinuria [16].

Safety data derived from post-marketing cohort studies of patients with chronic HBV infection are limited. Regarding Fanconi syndrome specifically, three case reports have been published in the existing literature [17,18]. Gracey et al. presented two cases; the first case was that of a 35-year-old male, a participant in the phase III trial for the efficacy of TDF who demonstrated an increase in serum creatinine with persistent hypouricemia along with glycosuria 48 months after TDF initiation [17]. A renal biopsy showed proximal tubulopathy with abundant giant mitochondria in electron microscopy [17]. The patient switched to entecavir and the normalization of renal tubular function was noticed 24 months later [17]. The second case concerned a 54-year-old patient under TDF treatment for HbeAg-negative chronic hepatitis who presented with high creatinine levels, aminoaciduria, glycosuria, low-grade proteinuria, hypophosphatemia and hypouricemia [17]. Due to the clinical suspicion of TDF-induced Fanconi syndrome, the treatment was switched to entecavir and tubulopathy was reversed, accompanied by a partial recovery of creatinine levels [17]. Furthermore, Hwang et al. published the case of a 44-year-old woman with chronic hepatitis B cirrhosis who presented 3 months after the initiation of tenofovir disoproxil with symptoms of severe hypokalemia, hypouricemia and metabolic acidosis. A renal biopsy was performed showing giant edematous mitochondria, leading to treatment modification with entecavir, leading to an improvement in biochemical parameters [18]. Table 2 summarizes the demographics and clinical characteristics of these cases.

Our case is the third case ever reported in the current literature where tenofovir-induced Fanconi syndrome was proven with a kidney biopsy. Regarding the severity of symptoms, it is the only case which presented with life threatening hypokalemia that required hemodialysis for the prevention of severe arrhythmias and acid–base disorders. The importance of a definitive diagnosis of this clinical entity lies in the total reversibility of the syndrome with causative agent withdrawal. Alternative agents such us tenofovir alafenamide and entecavir could ideally be used with a long-lasting, less nephrotoxic therapeutic result. Additionally, this is the case with the longest onset of renal impairment symptoms, as the patient had already been receiving tenofovir for the past 5 years since her diagnosis. However, some weaknesses of the case report are to be mentioned. These include the lack of a detailed medical history regarding the exact timing of the HBV diagnosis as well as the patient’s compliance to tenofovir disoproxil. While the patient was on active therapy with vitamin D supplements and NSAIDs, it was rather difficult to attribute severe bone disease and renal injury to proximal tubulopathy.

In comparison to tenofovir disoproxil, a prodrug, tenofovir alafenamide has been tested for efficacy and bone and renal safety as a rescue treatment in patients with hepatitis B in a randomized, double-blind, phase III clinical trial. Patients with chronic-HBeAg negative hepatitis B were randomized to receive either tenofovir disoproxil fumarate or alafenamide [3]. The patients receiving tenofovir alafenamide had a smaller decline in the estimated glomerular filtration rate compared with those receiving tenofovir disoproxil fumarate, although the change in serum creatinine was not significantly different at week 48 [3]. Since its approval, three case reports have reported renal injury related to tenofovir alafenamide [20]. One patient had an intentional overdose without tubular injury; another patient had evidence of chronic tubular injury induced by tenofovir in addition to acute kidney injury from other causes; and the third patient had evidence of tubular injury with tenofovir alafenamide, but only after starting a treatment regimen for hepatitis C that contained ledipasvir, which can increase tenofovir levels [20].

Another approach to prevent tenofovir-induced renal impairment includes the use of agents that could potentially prevent the toxic metabolite from entering proximal tubular cells or enhancing its exit. Probenecid, an inhibitor of hOAT1, is used to prevent cidofovir nephrotoxicity and may also protect from tenofovir [21]. However, 56% of patients had side effects ascribed to probenecid when used to prevent the proximal tubular toxicity of cidofovir, which was dose-limiting in 7% of patients [21]. Rosiglitazone, a peroxisome proliferator-activated receptor gamma agonist that induces the expression of many proximal tubular cell transporters, protected rats from tenofovir-induced renal failure and proximal tubular dysfunction [22]. However, concerns over the cardiovascular safety of rosiglitazone have led to its withdrawal from European markets. Clearly, more research is needed on nephroprotective strategies.

Proximal tubular cells are particularly sensitive to the toxic effects of tenofovir due to their unique set of cell membrane transporters that favor the entry of the drug. While the proposed molecular mechanisms for this injury remain unclear, routine testing for kidney function has been proposed to prevent the manifestations of renal injury. The 2017 clinical practice guidelines published by the European Association for the Study of the Liver propose that patients with chronic hepatitis B at high renal risk undergoing any NA therapy should be monitored via the observation of serum creatinine (eGFR) levels [23]. The frequency of renal monitoring can be every 3 months during the first year and every 6 months thereafter, if there is no deterioration. Closer renal monitoring is required in patients who develop acute renal injury with creatinine clearance below 60 mL/min or serum phosphate levels below 2 mg/dL [23]. On the other hand, the updated recommendations of the American Association for the Study of Liver Diseases on the treatment of chronic hepatitis B suggest that patients on active treatment with tenofovir disoproxil, serum creatinine, phosphorus, urine glucose and urine protein should be assessed before drug initiation and monitored at least once per year [24]. However, the quality of the evidence is considered to be low, introducing a high level of uncertainty for such clinical practices [24]. In our case, there was an annual monitoring of renal function in the context of the patient’s regular checkup, but no warning findings have been gradually observed so far.

## 4. Conclusions

To conclude, this is the third case of biopsy-proven tenofovir-induced Fanconi syndrome presented with the most extreme clinical presentation of renal impairment requiring hemodialysis. In this regard, more clinical research is required, firstly to identify the populations that are at higher risk of developing tenofovir renal toxicity, secondly to identify possible interactions with other drugs, and finally to detect early biochemical deterioration before clinical manifestations [19].

## Figures and Tables

**Table 1:** Patient’s laboratory test results at baseline.

Hb	14 g/dL	42–50%	Κ	1.8 mmol/L	3.5–5.1 mmol/L
Ht	42.2%	4.5–5.9 × 10^6^/μL	Νa	131 mmol/L	136–148 mmol/L
RBC	4.78 × 10^6^/μL	80–96/27–33/11.5–14.5	AST	25 U/L	5–40 U/L
MCV/MCH/RDW	90/29.9/15.5	4000–10,000/μL	ALT	16 U/L	5–40 U/L
WBC	26,300/μL	(40–70%/19–48%/2–10%)	CPK	130 U/L	5–170/L
(Neutrophils/Lymphocytes/Monocytes)	(98%/1.2%/0.6%)	(40–70%/19–48%/2–10%)	LDH	347 U/L	135–225 U/L
PLTS	201,000/μL	140,000–400,000/μL	Glucose	78 mg/dL	75–115 mg/dL
			Urea	64 mg/dL	18–50 mg/dL
			Creatinine	2.79 mg/dL	0.7–1.2 mg/dL
PT	13.4 s	10–15 s	tBil	0.28 mg/dL	0.3–1.2 mg/dL
aPTT	28.6 s	26–36 s	Uric acid	1.1 mg/dL	3–7 mg/dL
INR	1.22	0.8–1	P	0.9 mg/dL	2.5–4.5 mg/dL
Fib	5.7	1.8–3	ALP	111 U/L	40–129 U/L
D-Dimers	0.80		Ca	8.5 mg/dL	8.5–10.2 mg/dL
CRP	<0.33 mg/L	0–5 mg/L	Total protein	3.8 g/dL	6.4–8.4 g/dL
TSH/fT4/T3	1.58/12.70 < 0.62		albumin	2.4 g/dL	3.5–5 g/dL
Urinalysis			Urine P	426 mmol/L	0.97–1.45 mmol/L
PH	5.5		Urine K	87 mmol/L	0–10 mmol/L
Specific gravity	1020		Urine Cl	86 mmol/L	20–40 mmol/L
Protein	+1		Urine Na	85 mmol/L	0–20 mmol/L
WBC	1–2		Urine anion gap	86 mEq/L	0–<10 mEq/L
RBC	15–20		HBV	+HbsAg	
			HBV viral load	0 copies	
Glucose	+		ABGS		
24-h urine protein	2178 mg/day		PH	7.11	7.35–7.45
			Sat02	98%	80–100%
			pO_2_	113 mmHg	80–100 mmHg
			pCO_2_	18 mmHg	35–45 mmHg
			HCO_3_	6.6 mmol/L	22–28.0 mmol/L
			Glu	3.3 mmol/L	3.5–5.4 mmol/L
			lac	0.9 mmol/L	0.0–2.0 mmol/L
			K	1.1 mmol/L	3.7–4.7 mmol/L
			Na	128 mmol/L	14–17.5 g/dL
			Cl	112 mmol/L	101–110 mmol/L

**Table 2:** Cases of tenofovir disoproxil-induced Fanconi syndrome.

	Case No. 1 (2013; Gracey) [17]	Case No. 2 (2013; Gracey) [17]	Case No. 3 (2014; Vigano) [19]	Case No. 4 (2014; Vigano) [19]	Case No. 5 (2015; Hwang) [18]
Sex	Male	Male	Male	Male	Female
Age (years)	39	52	58	62	44
Origin	South-East Asian	Mediterranean	Italian	Italian	South-East Asian
Therapy	TDF 300 mg once daily	TDF 300 mg once daily	TDF 245 mg once daily	TDF 245 mg once daily	TDF 300 mg once daily
Indication	HBe-Ag (-) CHB	HBe-Ag (-) CHB	Hbe-Ag (-) CHB	Hbe-Ag (-) CHB	HBe-Ag (-) CHB
HBV DNA levels	110,000 IU/mL	6,400,000 IU/mL	<12 IU/mL	121,780 IU/mL	12,300 IU/mL
Comorbidities	Hypertension	Obesity, Dyslipidaemia, Hypertension, Sleep apnea	None	Hypertension	Low BMI, Diabetes
Onset	48 months	24 months	30 months	45 months	3 months
Cr_baseline_	96 μmol/L (EGFR: 81 mL/min/1.73 m^2^)	94 μmol/L (EGFR: 77 mL/min/1.73 m^2^)	0.90 mg/dL (EGFR: 89 mL/min/1.73 m^2^)	0.9 mg/dL (EGFR: 88 mL/min/1.73 m^2^)	1.03 mg/dL (EGFR: 58.2 mL/min/1.73 m^2^)
Cr_fanconi_	127 μmol/L (EGFR: 59 mL/min/1.73 m^2^)	135 μmol/L (EGFR: 51 mL/min/1.73 m^2^)	1.32 mg/dL (EGFR: 55 mL/min/1.73 m^2^)	3.35 mg/dL (EGFR: 18 mL/min/1.73 m^2^)	3.22 mg/dL (EGFR: 15.6 mL/min/1.73 m^2^)
Potassium_serium_	NA	NA	4.7 mEq/L	3.6 mEq/L	2.0 mEq/L
Uric Acid_serum_	0.21 mmol/L	0.08 mmol/L	NA	NA	2.5 mg/dL
Phosphate_serum_	0.8 mmol/L	0.68 mmol/L	2.0 mg/dL	1.7 mg/dL	2.6 mg/dL
Microglobinuria	NA	NA	NA	Yes	Yes
Proteinuria	0.6 g/24 h	0.2 g/24 h	0.05 g/24	0.3 g/24	Severe
Glycosuria	Yes	Yes	Yes	Yes	Yes
Osteoporosis (T-score)	NA	NA	Yes	No	NA
Biopsy	Proximal Tubular Injury	Not performed	Not performed	Not performed	Proximal Tubular Injury
Switch Agent	Entecavir 0.5 mg daily	Entecavir 0.5 mg daily	Entecavir 0.5 mg daily	Entecavir 0.5 mg every other day	Entecavir 0.5 mg daily
Time to tubular restoration	6 months	3 months	3 months	9 months	NA

## Data Availability

Data are contained within the article.
